# Smoking patterns during COVID-19: Evidence from Serbia

**DOI:** 10.18332/tid/148169

**Published:** 2022-05-31

**Authors:** Jovan Zubović, Aleksandar Zdravković, Olivera Jovanović

**Affiliations:** 1 Institute of Economic Sciences, Belgrade, Serbia

**Keywords:** Serbia, smoking intensity, COVID-19, smoking patterns

## Abstract

**INTRODUCTION:**

Recent research shows that in many countries, smokers who increased smoking during the COVID-19 pandemic outnumbered those who decreased it, despite higher exposure to COVID-19 severity and death. In this study, we compared changes in smoking behavior in the initial and later stages of the pandemic and factors associated with lower smoking intensity.

**METHODS:**

We conducted two survey rounds on representative samples, the first in the initial stage of the pandemic (11–19 May 2020) and the second in the latter stage (4–11 June 2021), in Serbia. Multinomial logistic regression was run to estimate associations between smokers’ characteristics and lower smoking intensity. In contrast to most studies that assess psychological factors affecting smoking in the pandemic, we focused on factual determinants of the change in smoking intensity.

**RESULTS:**

The share of smokers who claimed to smoke more increased significantly, from 22.7% in May 2020 to 35.2% in June 2021. The share of smokers who reported a decrease in smoking only slightly increased, from 11.7% to 13.3%. The persistence of the pandemic considerably increased intentions to cease smoking, from 26.3% to 40%. Higher education (adjusted odds ratio, AOR=2.11; 95% CI: 1.08–4.13), income fall (AOR=2.14; 95% CI: 1.11–4.11), occasional smoking (AOR=4.24; 95% CI: 2.26–7.96) and recovery from COVID-19 (AOR=2.38; 95% CI: 1.23–4.60) increased the odds to reduce smoking during the pandemic.

**CONCLUSIONS:**

Most smokers are unaware that consuming tobacco products increases exposure to health risks related to COVID-19, but those who get coronavirus infected tend to reduce smoking. This research highlights that tobacco control policy needs to be more proactive in creating public campaigns which demonstrate the severity of COVID-19 impact on smokers’ health. Such campaigns should mainly target those groups of smokers who are less able to curb their smoking intensity.

## INTRODUCTION

The outbreak of the COVID-19 pandemic posed a global threat to the world population’s physical and mental health. Over 1.8 million COVID-19 deaths were officially reported only in 2020, while the WHO assessment suggests at least 3 million deaths attributable to COVID-19 in 2020^1^. The COVID-19 pandemic also severely affected the general population’s mental health in terms of psychological distress, anxiety, and depression^2^. Smokers are particularly exposed to health risks, as smoking significantly increases the risk of COVID-19 progression, severity, and death^3,4^. Moreover, smoking cessation for one month or more is beneficial to reduce the risk of developing COVID-19 and its respective complications^5^.

Despite higher health risks, smokers who started, relapsed, or increased smoking during the pandemic outnumbered those who reduced or quit smoking in many countries, especially in high-income countries^6-13^. The studies on smoking behavior in low-and-middle-income countries during the pandemic are not so numerous, and outcomes are mixed: both net decrease^14,15^ and increase^16-18^ in smoking intensity are reported. Even in those countries where overall improvement in smoking behavior is documented, a significant share of smokers increased tobacco consumption. It is a matter of particular concern, as the duration of the pandemic is unknown and whether the end of the pandemic will contribute to the decline in smoking intensity.

The existing research on the change in smoking habits during the COVID-19 pandemic predominantly focused on the psychological factors associated with the shift in consumption of tobacco products and intentions to quit smoking. The empirical studies identified a wide range of stress-related psychological aspects to be associated with higher consumption of tobacco products, such as deterioration of mental health^19^, anxiety and depression^7,12^, loneliness and isolation^8,16^, concerns for someone else’s health^12^, or boredom^6,10,11^. Overall, there is strong empirical evidence that higher psychological distress and worsening psychological well-being are to blame for the higher intensity of smoking, especially in those countries that experienced harsh lockdowns. On the other hand, health concerns almost uniformly appear as the primary factor for reduced smoking or smoking cessation^14,20,21^.

In Serbia, the prevalence of tobacco use has been among the highest in Europe, even before the pandemic. This study aims to explore: 1) dynamic aspects of changes in smoking behavior regarding the initial and the later stage of the COVID-19 pandemic, and 2) factors associated with the overall decrease in smoking intensity during the COVID-19 pandemic.

## METHODS

### Study design

We applied multiple cross-sectional studies, based on two rounds of the survey on independent representative samples of the Serbian adult population aged 18–65 years, including smokers and non-smokers. We collected data through a telephone survey, being the most convenient way to examine participants under social distancing conditions. The 2020 survey (N=1002) was conducted 11–19 May 2020, several days after the government of Serbia initiated a lockdown to impede the spread of the COVID-19 virus. The 2021 survey (N=1402) was conducted 4–11 June 2021, when most of the population got used to living in pandemic conditions. Participation in the survey was voluntary and anonymous, and personal data were collected and processed in line with GDPR provisions.

### Measures and variables

The questionnaire is based on the survey of tobacco products consumption in Southeastern Europe^22^, which was implemented in cooperation with the University of Illinois Chicago as a component of the project ‘Accelerating Progress on Effective Tobacco Tax Policies in Low-and Middle-Income Countries’. The original questionnaire was modified to reflect the impact of the pandemic on subjects’ characteristics and smoking behavior.

The questionnaire used in the 2020 survey collected information on the subjects’ sociodemographic, financial and smoking characteristics, focusing on the change in smoking habits and economic well-being. A detailed explanation of the variables and metrics used in the first survey round has been published^18^. The questionnaire used in the 2021 survey was extended to collect information on health issues related to COVID-19, including vaccination status, personal recovery from COVID-19 and coronavirus infection cases among the household members. It is essential to mention that due to the multiple cross-sectional nature of the study, it was not possible to measure variables at the level of individual participants. Therefore, all variables are estimated at the aggregate level, separately for 2020 and 2021.

### Statistical analysis

Statistical analysis contains descriptive analysis and logistic regression. Descriptive statistics were used to present the current smokers’ characteristics and smoking habits separately for 2020 and 2021. We only consider changes in smoking behavior at the aggregate level, directly comparing descriptive statistics for 2020 and 2021.

Multinomial logistic regression was run to estimate associations between smokers’ characteristics (predictors) and changes in smoking intensity (dependent). The regression is solely based on the participants in the 2021 survey who were smokers. We could not run the regression for the dataset collected in the 2020 survey since information on some predictors was unavailable at the initial pandemic stage (for instance, vaccination status). The change in smoking intensity was simply measured by the question: ‘Do you smoke more/same/less than before the pandemic?’. We merged the response options ‘smoke more’ and ‘smoke same’ as we are particularly interested in factors associated with lower smoking intensity. The logistic regression results are presented as odds ratios (ORs) with 95% confidence intervals (CIs). A p<0.05 indicates statistical significance. The Stata version 16.0 was used to conduct the statistical analysis.

## RESULTS

### Baseline characteristics

Table 1 presents baseline characteristics of the current smokers’ baseline characteristics in the 2021 survey. The share of current smokers in the total sample is 40.1% (n=562). Female smokers were almost half (49.5%) of current smokers. Most of the smokers were aged 45–65 years (44.3%), tertiary educated (52.3%), full-time employed (62%) and lived in urban areas (60.7%). The number of unvaccinated smokers (51.1%) slightly exceeds vaccinated, whereas 29% of the total smokers recovered from COVID-19. The vast majority of the smokers (69.9%) lived in a household wherein at least one more member is a smoker, while 42.9% reported at least one household member infected by COVID-19. Around one-third of the smokers claimed higher spending on tobacco products since the pandemic (35.2%), more than those who reported higher spending on alcoholic beverages (12.6%).

**Table 1:** Characteristics of Serbian smokers

*Characteristics*	*Categories*	*2020 n (%)*	*2021 n (%)*
**Total**, n		420	562
**Sociodemographic**			
Gender	Female	217 (52.9)	278 (49.5)
	Male	193 (47.1)	284 (50.5)
Age (years)	18–24	45 (10.9)	65 (11.5)
	25–34	81 (19.8)	124 (22.1)
	35–44	90 (22.0)	124 (22.1)
	45–65	194 (47.3)	249 (44.3)
Education level	Secondary or less	254 (61.9)	265 (47.7)
	Tertiary	156 (38.1)	290 (52.3)
Type of settlement	Rural	119 (29.0)	221 (39.3)
	Urban	291 (71.0)	341 (60.7)
Employment status	Full-time	205 (50.9)	341 (62.0)
	Part-time	43 (10.7)	55 (10.0)
	Unemployed/out of labor force	155 (38.5)	154 (28.0)
**Financial**			
Income change since COVID-19 pandemic	Higher or same	235 (57.6)	375 (66.7)
	Lower	173 (42.4)	187 (33.3)
Spending on tobacco products since the COVID-19 pandemic	Less or same	N/A	364 (64.8)
	More	N/A	198 (35.2)
Spending on alcoholic beverages since the COVID-19 pandemic	Less or same/no expenses	N/A	491 (87.4)
	More	N/A	71 (12.6)
**Tobacco consumption**			
Smoking frequency	Regular	347 (84.6)	451 (80.3)
	Occasional	63 (15.4)	111 (19.7)
Tobacco product consumed	Cigarettes	335 (81.7)	458 (81.5)
	Tobacco (cut)	57 (13.9)	63 (11.2)
	Other	18 (4.4)	41 (7.3)
At least one more household member is a smoker	No	N/A	169 (30.1)
	Yes	N/A	393 (69.9)
The intensity of smoking since the COVID-19 pandemic	Less than before	48 (11.7)	74 (13.3)
	Same as before	268 (65.5)	336 (51.4)
	More than before	93 (22.7)	152 (35.2)
Intention to cease smoking since the COVID-19 pandemic	No	302 (73.7)	337 (60.0)
	Yes	108 (26.3)	225 (40.0)
**Health**			
Vaccinated	No	N/A	287 (51.1)
	Yes	N/A	275 (48.9)
Recovered from COVID-19	No	N/A	399 (71.0)
	Yes	N/A	163 (29.0)
At least one household member infected by the COVID-19	No	N/A	321 (57.1)
	Yes	N/A	241 (42.9)

When 2021 descriptive statistics on current smokers are compared to the 2020 survey, several changes in smoking habits are observed. The share of smokers who claimed to smoke more increased from 22.7% to 35.2%; 13.3% of the smokers reported a lower smoking intensity, slightly higher than 11.7% reported in 2020. Overall, the 2021 survey round results indicate a higher smoking intensity since the pandemic. On the other hand, the persistence of the pandemic motivated smokers to think about quitting smoking, as the intentions to cease smoking considerably increased from 26.3% to 40%. The other positive aspect is the decline of everyday smokers from 84.6% to 80.3%. The consumption structure of tobacco products mostly remained stable.

### The associations between characteristics of smokers and change in intensity of tobacco products consumption

Table 2 shows the multinomial logistic regression results examining the associations between change in intensity of tobacco products consumption and characteristics of smokers in 2021. Two of the characteristics presented in Table 1 were not included in the regression model: spending on tobacco products since the COVID-19 pandemic and intention to cease smoking since the COVID-19 pandemic. The former was not included as causality is running from the intensity of smoking to spending on tobacco consumption. The latter was not included as it captures forward-looking intentions.

**Table 2:** Factors identified with reporting to smoke less during the pandemic, logistic regression results, 2021 survey, Serbia

*Variable*	*Category*	*OR (95% CI)*	*AOR (95% CI)*
**Sociodemographic**			
Gender	Female (Ref.)		
	Male	0.88 (0.51–1.54)	0.79 (0.44–1.42)
Age (years)	18–24 (Ref.)		
	25–34	0.4 (0.16–1.01)*	0.37 (0.13–0.99)*
	35–44	0.76 (0.31–1.85)	0.76 (0.28–2.1)
	45–65	0.67 (0.3–1.52)	0.98 (0.38–2.5)
Education level	Secondary or less (Ref.)		
	Tertiary	1.6 (0.91–2.82)	2.11 (1.08–4.13)*
Type of settlement	Rural (Ref.)		
	Urban	1.02 (0.58–1.79)	0.83 (0.45–1.52)
Employment status	Full-time (Ref.)		
	Part-time	1.27 (0.53–3.06)	1.24 (0.48–3.21)
	Unemployed/out of labor force	0.78 (0.4–1.51)	0.61 (0.27–1.37)
**Financial**			
Income change since COVID-19 pandemic	Higher or same (Ref.)		
	Lower	1.65 (0.95–2.88)*	2.14 (1.11–4.11)*
Spending on alcoholic beverages since the COVID-19 pandemic	Less or same/no expenses (Ref.)		
	More	0.39 (0.16–0.97)*	0.22 (0.08–0.59)*
**Tobacco consumption**			
Smoking frequency	Regularly (Ref.)		
	Occasionally	3.17 (1.77–5.69)*	4.24 (2.26–7.96)*
Tobacco product consumed			
	Cigarettes (Ref.)		
	Tobacco (cut)	0.74 (0.32–1.7)	0.79 (0.26–2.38)
	Other	1.6 (0.6–4.25)	0.86 (0.32–2.27)
At least one more household member is a smoker	No (Ref.)		
	Yes	1.12 (0.61–2.07)	1.11 (0.55–2.22)
**Health**			
Vaccinated	No (Ref.)		
	Yes	0.9 (0.52–1.57)	0.94 (0.49–1.82)
Recovered from COVID-19	No (Ref.)		
	Yes	2.22 (1.25–3.92)*	2.38 (1.23–4.60)*
At least one household member infected by the COVID-19	No (Ref.)		
	Yes	1.57 (0.9–2.71)	1.1 (0.59–2.05)

AOR: adjusted odds ratio; adjusted for other predictors.

* p<0.05.

The adjusted odds ratios (AORs) indicate that six out of thirteen characteristics (Table 2) included in the logistic model are associated with lower intensity of tobacco products consumption. Among sociodemographic traits, gender, employment status, and type of settlement do not appear associated with smoking intensity. Smokers with tertiary education were two-times more likely to smoke less during the pandemic (AOR=2.11; 95% CI: 1.08–4.13, p<0.05). Regarding age, the odds to smoke less were lower in the group of smokers aged 25–34 years relative to the group of younger smokers (aged 18–24 years) being the reference category (AOR=0.37; 95% CI: 0.13–0.99 , p <0.05).

Variables depicting a change in the level and structure of the income of the Serbian smokers appear associated with lower smoking intensity. The smokers who suffered income fall during the pandemic were two-times as likely to smoke less (AOR=2.14; 95% CI: 1.11–4.11, p<0.05). An increase in spending on alcoholic beverages considerably declines the odds of lower smoking intensity (AOR=0.22; 95% CI: 0.08–0.59, p<0.01), indicating that consumption of alcohol and tobacco covariates in the same direction. Amidst health issues, vaccination and facing COVID-19 among household members did not affect the odds of a change in smoking intensity. On the other hand of a change in smoking intensity. On the other hand, smokers infected by the coronavirus had more than two-times higher odds of smoking less (AOR=2.38; 95% CI: 1.23–4.60, p<0.01). Regarding smoking habits, the occasional smokers were four-times more likely to reduce smoking during the pandemic (AOR=4.24; 95% CI: 2.26–7.96, p<0.001). The type of tobacco product consumed, and other smokers among household members, were not associated with a change in smoking intensity.

## DISCUSSION

The vast majority of studies on the change in smoking patterns during the COVID-19 pandemic indicate the double-edged pandemic’s impact on smoking intensity and cessation. The country-specific studies showed that the relation between smokers who increased smoking and those who decreased is not uniform and varies across countries. We contributed to the existing literature by measuring the number of smokers who increased/decreased their consumption of tobacco products and considering dynamic aspects of the change in smoking patterns during the pandemic. Our analysis revealed two opposite trends: the share of smokers who increased smoking intensity during the pandemic went up from 22.7% to 35.2% in parallel with intentions to cease smoking from 26.3% to 40%. The increase in the share of smokers who smoke more is probably related to the unanticipated persistence of the pandemic over 2021 and pandemic-related psychological distress and uncertainty about future financial and health well-being.

In this study, we merely focused on the behavioral, financial and physical health determinants of the change in smoking intensity rather than psychological determinants. We particularly examined characteristics of the smokers associated with improvement in smoking habits. The logistic regression analysis reveals that most sociodemographic factors do not affect the odds of less smoking during the pandemic. However, more educated smokers have higher odds to reduce smoking, similar to evidence that higher education is associated with smoking cessation^9^. Moreover, smokers aged 25–34 years are less likely to reduce smoking, in line with the finding that younger smokers have higher odds to quit smoking^7^.

The lower smoking intensity is significantly associated with income fall during the pandemic. It is in line with the theoretical premise that smoking prevalence declines with income fall due to the negative elasticity of demand for tobacco products concerning income, which is well documented in many empirical studies^23-27^. The increase in the price of cigarettes during the pandemic also reduced the affordability of tobacco products for smokers who suffered income falls. While an increase in alcohol consumption is less unfavorable than an increase in smoking, the strong association between those two variables is confirmed^12^, and therefore is a matter of grave concern for public health.

The occasional smokers are more likely to reduce their smoking intensity, which is expected since they are less addicted and usually smoke at social events that were banned or restricted by the containment measures. Type of the tobacco product consumed appears as an irrelevant predictor of the change in smoking habits. It is also in line with results from our previous study that occasional smokers are more likely to quit smoking, while the type of tobacco product does not affect smoking cessation odds^18^. The consumption of tobacco products by the other household members is not associated with smoking intensity, which is somewhat opposite to finding that not living with a smoker increases the odds of smoking cessation^9^.

The health concerns were emphasized as the most influential determinants contributing to smoke reduction during the pandemic. We contribute to the literature by opting for tangible descriptors of the smokers’ health concerns related to the pandemic, including vaccination, recovery from COVID-19 or cases of coronavirus infection within the household. It turns out that those smokers infected by COVID-19 are more capable of reducing their consumption of tobacco products. At the same time, other health factors do not appear as predictors of smoking intensity. A study^28^ assessed an association between COVID-19 vaccination and smoking status, but no previous research examines associations of change in smoking habits with immunization.

### Limitations

The study has some limitations and room for further improvement. We did not conduct longitudinal research; thus, dynamic changes in smoking behavior are presented only at the aggregate level, not at the level of individuals. Changes in smoking intensity are only qualitatively addressed; therefore, the study lacks quantitative information on increasing or decreasing tobacco products consumption. Eventually, we did not control the impact of health factors other than COVID-19 related, such as a history of chronic or acute illnesses.

### Implications

Overall, our study shows that the COVID-19 pandemic has worsened smoking behavior. Therefore, it is of utmost importance for policymakers to consider these findings and increase efforts to promote tobacco control policies during the pandemic, raising smokers’ awareness about the severity of COVID-19 impact on smokers’ health. The finding on the negative association between smoking frequency and change in smoking intensity is a strong argument supporting the smoking ban in bars and restaurants, being the location where occasional smokers usually consume tobacco products (in Serbia, smoking in bars and restaurants is still not banned). Further research on the subject will aspire to overcome existing limitations by extending the questionnaire to include additional factors that may predict smoking behavior and quantitative measures of change in smoking intensity.

## CONCLUSIONS

The survey on the national representative sample showed that smokers who increased their consumption of tobacco products outnumbered those who smoked less relative to their pre-COVID-19 smoking levels. The reduced smoking intensity during the pandemic was associated with higher education, income fall, occasional smoking, and recovery from COVID-19. This research highlights that tobacco control policy needs to be more proactive in creating public campaigns which demonstrate the severity of COVID-19 impact on smokers’ health. Such campaigns should mainly target those groups of smokers who cannot restrain their smoking intensity. Also, it turns out that pandemic conditions hinder tobacco consumption among occasional and young smokers, probably due to reduced social interactions. This unique opportunity should be utilized to intensify smoking cessation campaigns for occasional and young smokers before they develop long-term addiction.

## Data Availability

The data supporting this research are available from the authors on reasonable request.
